# The Treatment of Epilepsy

**Published:** 1850-06

**Authors:** 


THE TREATMENT OF EPILEPSY.—DR. CHENEAU has presented to the Academy of Sciences, Paris, a memoir on the treatment of epilepsy, which is well worthy of perusal. He desires to prove that the disease is curable by medicine, resting his principal mode of arresting its progress upon the judicious employment of digitalis. The cases that have proved the most inveterate have yielded to a perseverance in the use of this remedy for a period of six or eight months. He has submitted six instances to the Academy, in which he has been successful ; the first occurred at the Bicetre, under the care of Dr. Voisin. A young man, aged twenty, had been epileptic, it is suppposed from fright, from his fourteenth year ; two months treatment restored him to health. The second was one in which the disease, produced at the age of thirteen, by fright, had lasted till the individual had attained his forty-second year. The treatment commenced the 20th of April, 1847, and after the 16th of June in the same year he had no attacks, which before had appeared, it is true, at only very long intervals ; as many as six years having at one time elapsed without any access of paroxysm. The third case is certainly one as singular as any that has been registered upon the rolls of medical science. A young lady, thirteen and a half years old, had been subject for several years, to the disease, which had at length brought on idiocy and paralysis of half the body ; the paroxysms were not very frequent, but were of great violence, frightening the persons who nursed her, the countenance wearing a purple and almost a black hue, which sometimes lasted twelve hours after the fit had ceased ; the hemiplegia, which was of the right side, prevented the movement of the limbs, and partially affected sensation. The treatment was commenced on the 4th of July, 1846, and by the month of January in the following year, the epileptic fits altogether ceased. A year has elapsed since the young lady has been able to go on with her education, and she is also able to run in the garden and to amuse herself with gymnastic exercises. The fourth noticed is that of a yonug girl of ten years of age, upon whom epilepsy supervened after fright ; it had lasted two years, but soon yielded to the usual remedy of Dr. Cheneau. The fifth case was that of a patient in the Bicetre ; he was, when placed under the treatment, sixteen years of age, and had been afflicted by the malady since he was five years old. At first, the paroxysms were but slight ; he suddenly turned himself mechanically round any object immediately in his neighborhood ; this lasted about a minute. As the disease advanced, he had convulsions, and the fits became very frequent. The remedies were commenced on the 2d of October, 1847, and in January, in the following year, was his last attack. Since that time his health has become perfectly established. The last case carries with it the same interest as the preceding ones. A boy, of ten years of age, after having been for three years subject to frequent fits, once as many as fourteen in twenty-four hours, was subjected to the use of the digitalis, and at the end of two months was an instance of the excellence of the system pursued by Dr. Cheneau. The essay is well written, and certainly demonstrates that digitalis, properly combined, has cured epilepsy, even when complicated with paralysis and idiotism ; that the cure has not been confined to youth, but has been decided even at an advanced age, and that the time occupied has not been of great duration. An incidental subject of discussion has been the subject of the sudden paleness of the face, which has been noted down by the nosologists as one of the symptoms of epilepsy. One of the characteristics, as given by Georget, in the "Dictionnaire de Medicine," is, "extreme pallor of the face, suddenly coming on towards the end of the fit, succeeding to a redness more or less intense which previously existed." In the hospital of the Bicetre, one hundred and twenty fits have been watched with the most scrutinizing care, and this state has never once presented itself ; on the contrary, the redness came on during the paroxysm, continued throughout the convulsions, and often lasted for some time afterwards. On no one occasion was the sudden paleness observed.

M. Delasiauve, one of the physicians to the Bicetre, is investigating with great attention the resources which we possess for the cure of epilepsy ; his observations on the employment of sedatives are of considerable value. To valerian he gives the first place, though he does not speak of it quite in as sanguine language as did Tissot ; a decoction of valerian given in doses of two wine glasses full, morning and evening, have produced a radical cure, but the medicine requires to be persevered in for a considerable length of time, otherwise it is of little avail ; assafœtida decidedly moderates the violence of the access, but it does not seem to produce the same permanent good effect. The hydrocyanate of iron isfound in some instances very beneficial ; belladonna and digitalis are each serviceable, but Pluvrey of Lille prefers a combination of the two. The root of artemisia is occasionally useful ; of liquid ammonia, according to the formula prepared by Martinet, he has some good reason to speak, and will shortly give the results of his experience ; camphor is found in those cases where the reproductive system is in a high state of excitement, as not unusually occurs in epileptics, to be of remarkable service ; zinc, musk, castor, ambergris, have but little curative power ; preparations of copper are to be but little confided in ; nitrate of silver has lost the high character it once obtained ; sulphate of quinine has also fallen into disrepute. He enters very minutely into the subject of the diet and exercise of the patient, preferring vegetable to animal food. It is to be regretted that the series of papers which this observant practitioner had prepared for the press are for the present suspended, for want of that encouragement which would have been at another period given to inquiries of such deep moment.—*Journal of Insanity.*

